# Doffing personal protective equipment in times of COVID-19

**DOI:** 10.47626/1679-4435-2021-605

**Published:** 2021-04-30

**Authors:** Gilcélia Correia Santos Bernardes, Ana Paula Nogueira Godoi, Nívea Aparecida de Almeida, Leilismara Sousa Nogueira, Melina Barros Pinheiro

**Affiliations:** 1 Universidade Federal de São João del-Rei, Campus Centro-Oeste Dona Lindu, Divinópolis, MG, Brazil

**Keywords:** COVID-19, personal protective equipment, health personnel, occupational health, occupational exposure

## Abstract

COVID-19, a disease caused by a coronavirus (SARS-CoV-2), has worried health authorities in Brazil and worldwide because of its high infectivity and rapid spread. Within this context, health care workers are at greater risk of infection for being in close contact with patients, which is inherent to their work activities. To reduce the risk, protective measures must be adopted and personal protective equipment is essential. However, the process of removing personal protective equipment, named doffing, is as important as its correct use and can be a source of contamination for workers, especially when equipment is lacking in the market and lifespan is increased. Therefore, this review aimed to discuss the process of doffing personal protective equipment and its correct sequence based on data available in the literature.

## INTRODUCTION

Coronavirus disease 2019 (COVID-19), initially named 2019-n-CoV, caused by the severe acute respiratory syndrome coronavirus 2 (SARS-CoV-2), appeared in the city of Wuhan, province of Hubei, People’s Republic of China. It has spread across the world and was classified as a pandemic on March 11, 2020, by the World Health Organization (WHO).^1-4^

The extent of COVID-19 infections follows an upward curve, and the WHO estimates that by July 21, 2020, 14,562,550 people had been infected and 607,781 deaths had been recorded worldwide.^5^ Brazil ranks second in the world in incidence of infections, after the United States, with a total of 2,159,654 cases, 81,487 deaths, mortality of 38.8 per 100 thousand population, and 1,465,970 recovered cases up to July 21, 2020.^2^

A major concern for all authorities during the pandemic is the rapid spread of COVID-19, caused by easy transmission. Because of the nature of their professional activity, which requires close contact with patients, front-line health care workers are at greater risk of contamination. For such reason, protective measures must be adopted to reduce the risk of contamination, and personal protective equipment (PPE) occupies a prominent position.^6^

Correct use of appropriate PPE is as important as its safe removal, since studies show that doffing is one of the most critical moments for worker contamination.^7,8^ Therefore, this review aims to discuss the process of doffing PPE and its correct sequence based on data available in the literature.

### RISK OF HEALTH CARE WORKER CONTAMINATION

Health care workers are required to carry out their work activities in close contact with patients, especially those on the front line. Thus, the risk of contamination is high for them.^9^

A study conducted by Ye et al.^10^ demonstrated that a hospital environment can be contaminated during care for patients with COVID-19, and several items of daily use can become sources of virus spread, such as computers, handles, keyboards, PPE, and printers. In such cases, when contamination occurs in the workplace and because of work activities, COVID-19 can be considered an occupational disease.

An occupational disease, or work-related disease, according to Brazilian Law no. 8,213 of July 24, 1991, is defined as a disease “contracted or triggered as a result of engagement in a particular work activity and included in the list prepared by the Brazilian Ministry of Labor and Social Security” [free translation.^11^

It remains unknown to what extent constant exposure affects the health of workers, and the number of infected workers remains uncertain; however, the International Council of Nurses (ICN) gathered data from national associations and reported that around 90 thousand workers had been infected until the report was published on May 6, 2020.^12^ According to the Brazilian Federal Nursing Council (Conselho Federal de Enfermagem, Cofen), 28,095 cases of COVID-19 had been reported among nursing workers, including 293 deaths due to COVID-19, in Brazil up to July 21, 2020.^13^

Therefore, health care workers, especially those on the front line to combat COVID-19, must strictly follow the established protocols and protective measures^4^ in an effort to reduce the risk of contamination, since contact with patients is often unavoidable. Within this context, use of PPE is recommended.

### PPE

PPE, according to Brazilian Regulatory Standard no. 6, is defined as “any individual device or product used by the worker and designed to protect against risks that are likely to threaten health and safety at work” [free translation].^14^ In health care settings, especially during the pandemic, recommendation is to wear a gown, surgical mask, N95/PFF2 respirator or equivalent, goggles and/or face shield, cap, and gloves, according to the risk involved in each professional activity.^15^

Employers are responsible for providing appropriate PPE according to the risks of each professional activity, for guiding and training workers on proper use, storage, and conservation, as well as for providing replacement when equipment is damaged.^14^ However, there is a current lack of PPE because of high world demand, thus requiring a more rational use without jeopardizing the health of workers and their patients.^16^

It is worth noting that wearing PPE by itself does not provide workers with full protection, as correct positioning and good conditions of PPE are required. Also, the process of PPE removal, named doffing, is as important as its correct use.

### THE RISK OF INCORRECT DOFFING

Wearing PPE and then doffing it correctly minimizes the chance of health care workers being contaminated by infectious diseases while they work and spreading pathogens.

During the Ebola outbreak, the importance of doffing became even more evident.^17^ Despite wearing the recommended PPE, two nurses in Texas, United States, were contaminated while they were treating a patient with Ebola in an intensive care unit, suggesting that contamination had occurred during the doffing sequence.^18^ Thus, North American health authorities intensified protective measures for workers treating patients with suspected Ebola and determined the use of N95/PFF2 respirators, instead of surgical masks, and face shields. They also recommended the presence of a trained observer to supervise each step of donning and doffing sequences.^19^

A study conducted by Tomas *et al*.^20^ assessed the risk of infection during PPE removal using a bacteriophage MS2 (a nonpathogenic, nonenveloped RNA virus) together with a fluorescent lotion. For 2 weeks, employees from four hospitals were invited to participate in the study, which found that contamination was frequent during removal of gloves and gowns. Based on the results, training was provided and resulted in reduced contamination during doffing sequence.^20^ This finding suggests that training focused on correct use and removal of PPE in health care settings can help reduce the risk of contamination and should be frequent.

Mitchell*et al.*^21^assessed the behavior of health care workers who were treating patients with fever and respiratory symptoms. Regarding hand hygiene, 26% of workers performed it after removing their gloves, 46% after removing their gown, and 57% after removing their mask or eye protection. Regarding doffing sequence, only 54% of workers removed their PPE correctly.^21^

Cases of infection with Middle East respiratory syndrome virus in hospital settings accounted for more than 40% of infections reported in Abu Dhabi, United Arab Emirates. Contamination generally occurred before patient diagnosis, and self-reported use of PPE among workers was contradictory.^22^

A study conducted by Varia *et al*.^23^demonstrated how a single patient infected with SARS-CoV caused an outbreak in a hospital in Toronto, Canada. Of the infected patients, 36.7% were hospital employees, 14.1% were patients, 14.1% were visitors, and 29.6% were household contacts.^23^

The risk of SARS-CoV-2 contamination during PPE removal also exists, since studies have demonstrated that high concentrations of viral RNA are present in PPE removal rooms.^24^ Therefore, health care workers performing aerosol-generating procedures in patients with confirmed or suspected COVID-19 should be very careful to avoid contamination during PPE removal.^6^

The doffing sequence is so critical that international institutions, such as the Centers for Disease Control and Prevention (CDC), as well as national institutions, such as Cofen, have prepared guides for correctly donning and doffing PPE.^19,25^ However, it is extremely important that health care workers receive appropriate training, which is an employer responsibility.^14^

**Figure 1:**
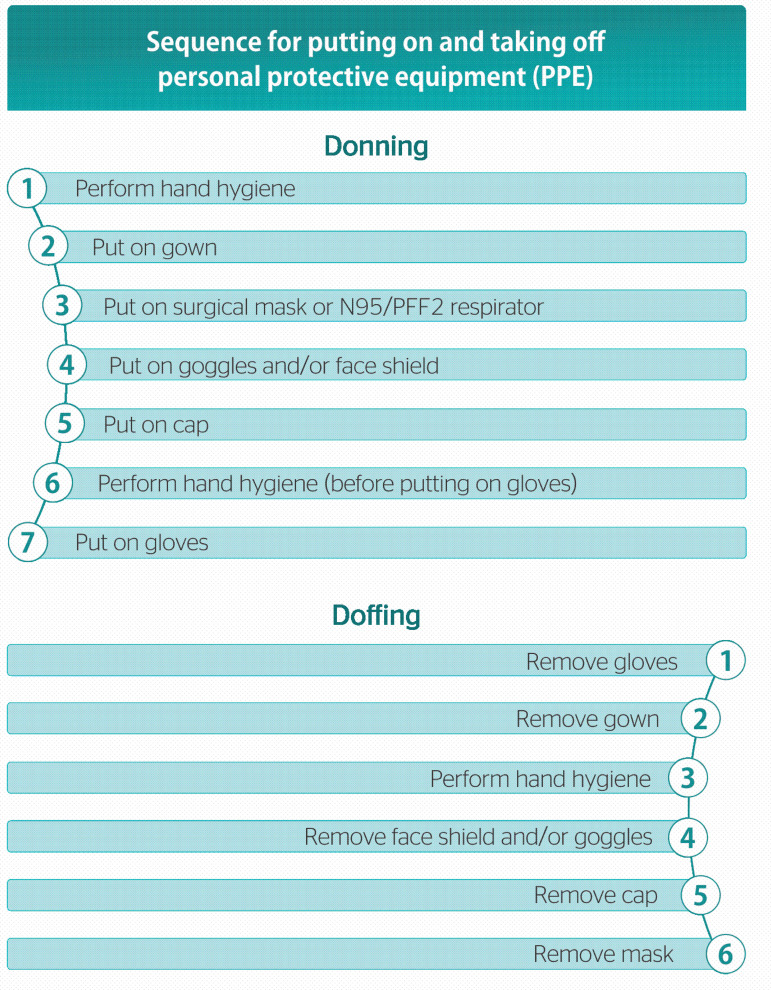
Sequence for putting on and taking off personal protective equipment (PPE).

The CDC and Cofen recommendations for putting on PPE are as follows (Figure 1): 1) perform hand hygiene with soap and water or 70% alcohol solution; 2) put on gown and tie its straps on your back; 3) put on N95/PFF2 respirator or surgical mask; 4) put on goggles or face shield; 5) perform hand hygiene before putting on gloves; 6) put on gloves. Cofen also recommends the use of a cap in aerosol-generating procedures, which should be put on after goggles or face shield.

PPE removal, in turn, should follow this sequence: 1) remove gloves, being careful not to touch the front; 2) remove gown; 3) perform hand hygiene with soap and water or 70% alcohol solution; 4) remove goggles or face shield; 5) remove mask, not touching the front. When wearing a cap or mask, remove it after removing the gown.^19,25^

## FINAL CONSIDERATIONS

Even in critical periods such as that of the COVID-19 pandemic, health and safety of health care workers must be prioritized and deserve due attention especially because of the high risk of infection inherent to their work activities. Therefore, it is of utmost importance that they have appropriate PPE available and that they are guided on its correct use, especially in relation to the doffing sequence, which is largely responsible for infections in workers.

Contamination of health care workers while carrying out their work activities is considered an occupational disease and, in addition to putting their families and other workers at risk, can impact the quality of services, since their absence is necessary and may even culminate in their death.

## References

[1] Zhu N, Zhang D, Wang W, Li X, Yang B, Song J (2020). A novel coronavirus from patients with pneumonia in China, 2019. N Engl J Med.

[2] Brasil, Ministério da Saúde (2020). Painel Coronavírus.

[3] World Health Organization (WHO) (2020). WHO Director-General's opening remarks at the media briefing on COVID-19 - 11 March 2020.

[4] Organização Pan-Americana da Saúde (OPAS) Folha informativa COVID-19 - Escritório da OPAS e da OMS no Brasil.

[5] World Health Organization (WHO) (2020). WHO Coronavirus Disease (COVID-19) Dashboard.

[6] Lockhart SL, Duggan LV, Wax RS, Saad S, Grocott HP (2020). Personal protective equipment (PPE) for both anesthesiologists and other airway managers: principles and practice during the COVID-19 pandemic. Can J Anaesth.

[7] Kwon JH, Burnham CD, Reske KA, Liang SY, Hink T, Wallace MA (2017). Assessment of healthcare worker protocol deviations and self-contamination during personal protective equipment donning and doffing. Infect Control Hosp Epidemiol.

[8] McLaws M-L, Chughtai AA, Salmon S, MacIntyre CR (2016). A highly precautionary doffing sequence for health care workers after caring for wet Ebola patients to further reduce occupational acquisition of Ebola. Am J Infect Control.

[9] Siegel JD, Rhinehart E, Jackson M, Chiarello L (2007). 2007 Guideline for Isolation Precautions: Preventing Transmission of Infectious Agents in Health Care Settings. Am J Infect Control.

[10] Ye G, Lin H, Chen S, Wang S, Zeng Z, Wang W (2020). Environmental contamination of the SARS-CoV-2 in healthcare premises: an urgent call for protection for healthcare workers. J Infect.

[11] Brasil (1991). Lei nº 8.213, de 24 de julho de 1991. Dispõe sobre os Planos de Benefícios da Previdência Social e dá outras providências.

[12] International Council of Nurses (ICN) (2020). ICN calls for data on healthcare worker infections and deaths.

[13] Conselho Federal de Enfermagem (Cofen) (2020). Profissionais infectados com COVID-19 informado pelo serviço de saúde.

[14] Brasil, Ministério do Trabalho (1978). Portaria nº 3.214, de 08 de junho 1978. Aprova as Normas Regulamentadoras - NR - do Capítulo V, Título II, da Consolidação das Leis do Trabalho, relativas a Segurança e Medicina do Trabalho.

[15] Brasil, Agência Nacional de Vigilância Sanitária (Anvisa) (2020). Nota técnica GVIMS/GGTES/ANVISA Nº 04/2020.

[16] World Health Organization (WHO) (2020). Rational use of personal protective equipment for coronavirus disease (COVID-19) and considerations during severe shortages.

[17] Fischer II WA, Hynes NA, Perl TM (2014). Protecting health care workers from Ebola: personal protective equipment is critical but is not enough. Ann Intern Med.

[18] Liddell AM, Davey Jr RT, Mehta AK, Varkey JB, Kraft CS, Tseggay GK (2015). Characteristics and clinical management of a cluster of 3 patients with Ebola virus disease, including the first domestically acquired cases in the United States. Ann Intern Med.

[19] Centers for Disease Control and Prevention (CDC) (2020). Using personal protective equipment (PPE).

[20] Tomas ME, Kundrapu S, Thota P, Sunkesula VC, Cadnum JL, Mana TS (2015). Contamination of health care personnel during removal of personal protective equipment. JAMA Intern Med.

[21] Mitchell R, Roth V, Gravel D, Astrakianakis G, Bryce E, Forgie S (2013). Are health care workers protected? An observational study of selection and removal of personal protective equipment in Canadian acute care hospitals. Am J Infect Control.

[22] Hunter JC, Nguyen D, Aden B, Al Bandar Z, Al Dhaheri W, Elkheir KA (2016). Transmission of Middle East respiratory syndrome coronavirus infections in healthcare settings, Abu Dhabi. Emerg Infect Dis.

[23] Varia M, Wilson S, Sarwal S, McGeer A, Gournis E, Galanis E (2003). Investigation of a nosocomial outbreak of severe acute respiratory syndrome (SARS) in Toronto, Canada. CMAJ.

[24] Liu Y, Ning Z, Chen Y, Guo M, Liu Y, Gali NK (2020). Aerodynamic analysis of SARS-CoV-2 in two Wuhan hospitals. Nature.

[25] Conselho Federal de Enfermagem (Cofen) (2020). Orientações sobre a colocação e retirada dos equipamentos de proteção individual (EPIs).

